# Intermetallic Pd_3_*X* (*X*= Ti and Zr) nanocrystals for electro-oxidation of alcohols and formic acid in alkaline and acidic media

**DOI:** 10.1080/14686996.2020.1789437

**Published:** 2020-08-24

**Authors:** Rajesh Kodiyath, Gubbala V. Ramesh, Maidhily Manikandan, Shigenori Ueda, Takeshi Fujita, Hideki Abe

**Affiliations:** a Center for Green Research on Energy and Environmental Materials, National Institute for Materials Science, Tsukuba, Ibaraki, Japan; b Department of Chemistry, Chaitanya Bharathi Institute of Technology (A), Hyderabad, Telangana, India; c Synchrotron X-ray Station at SPring-8, National Institute for Materials Science, Sayo, Hyogo, Japan; d WPI Advanced Institute for Materials Research, Tohoku University, Sendai, Japan

**Keywords:** Pd_3_Ti nanoparticles, Pd_3_Zr nanoparticles, intermetallic compounds, electro-oxidation, fuel cells, 205 Catalyst / Photocatalyst / Photosynthesis, 106 Metallic materials, 206 Energy conversion / transport / storage / recovery, 207 Fuel cells / Batteries / Super capacitors, 301 Chemical syntheses / processing, 502 Electron spectroscopy, 503 TEM, STEM, SEM

## Abstract

Two highly active and stable Pd-based intermetallic nanocrystals with early d-metals Pd_3_Ti and Pd_3_Zr have been developed. The nanocrystals are synthesized by co-reduction of the respective salts of Pd and Ti/Zr. Hard X-ray photoemission Spectroscopy (HAXPES) analysis of the nanocrystals indicates that the electronic properties of Pd are modified significantly, as evident from the lowering of the d-band center of Pd. The intermetallic nanocrystals dispersed in Vulcan carbon, Pd_3_Ti/C and Pd_3_Zr/C, exhibit improved electrocatalytic activity towards methanol and ethanol oxidation in an alkaline medium (0.5 M KOH), compared to those of commercially available catalysts such as Pd/C, Pt/C, and Pt_3_Sn/C. In addition, Pd_3_Ti/C and Pd_3_Zr/C show significantly higher activity towards the oxidation of formic acid in an acidic medium (0.5 M H_2_SO_4_), compared to those of Pd/C and Pt/C. The modification of the d-band center of Pd as a result of the alloying of Pd with the early d-metals Ti and Zr may be responsible for the enhanced catalytic activity.

## Graphical Abstract

**Figure:**
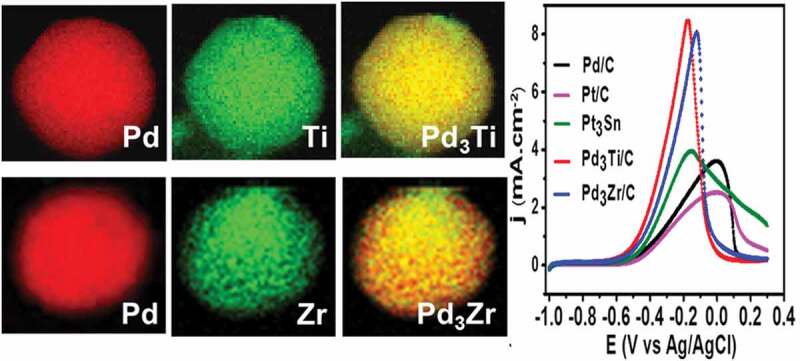

## 1. Introduction

Direct liquid fuel cells, such as direct alcohol fuel cells (DAFC) and direct formic acid fuel cells (DFAFC), have emerged as a promising energy conversion technology because of their high efficiency and low pollution [1–3]. Pt and Pt-based alloys are the most extensively studied and best catalysts for DAFC and DFAFC [4–8]. However, the use of Pt is limited due to its high cost of Pt, slow reaction kinetics, low stability, and poisoning by CO, which is formed during the electro-oxidation of alcohols or acids [9–13]. Therefore, developing low-cost catalysts with higher activity and stability is of paramount importance for the commercialization of DAFC or DFAFC.

Palladium has attracted increasing attention due to its lower cost, greater abundance, and excellent electrocatalytic activity towards the oxidation of small organic molecules, particularly in alkaline media [14–19]. Alloying Pd with other metals, such as Ag, Au, Sn, Pb, Ni, Cu, etc., is an efficient way to improve the activity of Pd by modifying the structural and electronic properties of the active sites [16,20–30]. Density functional theory (DFT) calculations suggest that the electronic properties of Pd (d-band center) can be modified or even matched with those of Pt by alloying the former with early d-metals such as Ti, Zr, and Ta [31,32]. Thus, alloys of Pd with early d-metals are expected to exhibit performances close to or even better than that of Pt in various catalytic reactions. However, developing such alloys is challenging due to the highly oxyphilic nature of the early d-metal precursors [33–35]. Herein, we present our attempt to develop a new class of intermetallic nanocrystals consisting of Pd and early d-metals, or Pd_3_*X* (where *X* = Ti and Zr), in addition to detailed characterization of these catalysts. The Pd_3_*X* nanocrystals are demonstrated to be efficient catalysts for the oxidation of methanol and ethanol in an alkaline medium. These catalysts also exhibit enhanced catalytic activity toward the oxidation of formic acid, promoting the favorable direct dehydrogenation pathway in an acidic medium. HAXPES measurements suggest that the d-band center of Pd is lowered. The modification of the electronic properties of Pd may be responsible for the enhanced activity of Pd_3_*X* nanocrystals in both alkaline and acidic media.

## 2. Experimental details

### 2.1 Reagents

We used anhydrous palladium (II) acetate, (Aldrich), anhydrous titanium(IV) chloride tetrahydrofuran complex (TiCl_4_ · 2THF, Aldrich,97%), zirconium (IV) chloride (ZrCl_4_, Aldrich, 99%), diglyme (anhydrous, 99.8%, Aldrich), LiBH(C_2_H_5_)_3_ (super-hydride, 1 M in THF, Aldrich), hexane (anhydrous, 95%, Aldrich), acetonitrile (99.8%, Aldrich), sodium metal (Aldrich) and naphthalene.

### 2.2. Synthesis of sodium naphthalide

Sodium naphthalide solution was prepared by dissolving 22.9 mg of metallic sodium and 129.4 mg of naphthalene in dry diglyme. The reaction mixture was stirred overnight under an argon atmosphere.

### 2.3. Synthesis of Pd_3_Ti/C NPs

Intermetallic Pd_3_Ti and NPs were synthesized by co-reduction of the metal precursors in diglyme.Palladium (II) acetate (0.13 mmol) and TiCl_4_ · 2THF (0.043 mmol) were weighed and transferred to a round-bottom flask containing the strong reducing agent sodium naphthalide. 54 mg of Vulcan carbon was added to the reaction mixture. The reaction mixture was then transferred to a stainless-steel pressure vessel and heated at 200°C in an oil bath for 2 h under an argon pressure of 0.5 MPa [34,35]. The product was then transferred to a centrifuge tube under an argon atmosphere. The precipitate was separated from diglyme by centrifuging at 6000 rpm for 5 min. The product was washed several times with hexane and acetonitrile to remove the byproducts, and then dried under vacuum for 1 h. The washing solvents were carefully selected to minimize interaction with the oxyphilic metal and hence, leaching or dissolution of the metal, resulting in a non-uniform composition. The as-prepared product was annealed at 1000°C for 15 h under vacuum to achieve the desired intermetallic Pd_3_Ti phase.

### 2.4. Synthesis of Pd_3_Zr/C NPs

Intermetallic Pd_3_Zr NPs were synthesized by co-reduction of the metal precursors in diglyme.Palladium (II) acetate (0.33 mmol) and ZrCl_4_ (0.11 mmol) were weighed in a stainless-steel pressure vessel. Then, 30 ml of diglyme were added to the vessel, and the mixture was stirred for 20 min to dissolve the reactants. Next, 1 ml of super-hydride was added to the reaction mixture, which was heated at 200°C in an oil bath for 2 h under an argon pressure of 0.5 MPa [34,35]. The product was then transferred to a centrifuge tube under an argon atmosphere. The precipitate was separated from diglyme by centrifuging at 6000 rpm for 5 min. The product was washed several times with hexane and acetonitrile to remove the byproducts and dried under vacuum for 1 h. The as-prepared product was then mixed with Vulcan carbon to form Pd_3_Zr/C. The as-prepared product Pd_3_Zr/C was annealed at 1000°C for 15 h under vacuum to obtain intermetallic Pd_3_Zr/C.

### 2.5 Synthesis of bulk Pd_3_Ti and Pd_3_Zr

Polycrystalline bulk samples of intermetallic Pd_3_Ti and Pd_3_Zr were synthesized with an arc furnace in a pure Ar atmosphere (99.9999%). Prior to the synthesis, the arc furnace was evacuated to a vacuum level lower than 10 mPa and back-filled with pure Ar. All of the starting materials were purchased from Furuya Kinzoku Co. An aliquot of 1 g of Pd powder (99.9%) was pelletized with a stainless-steel die and melted into an ingot using an arc furnace. Ti/Zr (ingot, 99%) was used as received. The ingots of Ti/Zr and Pd were weighed such that the molar ratio was Ti/Zr:Pt = 1:3 and melted together in an arc furnace to obtain the desired intermetallic Pd_3_Ti/Pd_3_Zr. The product was finally annealed in vacuum at 1000°C for 72 h.

## 3. Characterization

### 3.1. *Powder X-ray diffractometry (*p*XRD)*

*p*XRD measurements were performed using Cu Kα radiation (Panalytical X’Pert PRO; λ = 0.1548 nm) in the range of diffraction angles from 20 to 100 degrees, with increments of 0.02 degrees. An obliquely finished Si crystal (non-reflection Si plate) was used as a sample holder to minimize the background.

### 3.2. Hard X-ray photoemission spectroscopy (HAXPES)

HAXPES measurements were performed using X-rays with a photon energy of 5.95 keV, at the undulator beamline BL15XU of SPring-8, Japan. Samples for the HAXPES measurements were prepared by mixing the sample solution (in THF) with carbon black (Vulcan XC-72, Cabot Co. Ltd.) to avoid charging effects. 10 µl of the sample was dropped onto a carbon substrate (Nilaco Co., Ltd.) and dried under vacuum. The core-level states of the samples were examined at room temperature in ultrahigh vacuum (UHV) using a hemispherical electron energy analyzer (VG SCIENTA R4000). The total energy resolution was set to 220 meV. The binding energy was referenced to the Fermi edge of an Au thin film.

### 3.3. Transmission electron microscopy

A 200 kV transmission electron microscope (TEM and/or STEM, JEM-2100 F, JEOL) was used. It was equipped with two aberration correctors (CEOS GmbH) for the image- and probe-forming lens systems and an X-ray energy-dispersive spectrometer (JED-2300 T, JEOL) for compositional analysis. Both the aberration correctors were optimized to realize point-to-point resolutions of 1.3 and 1.1 Å for TEM and scanning transmission electron microscopy (STEM), respectively. A probe convergence angle of 29 mrad and a high-angle annular-dark-field (HAADF) detector with an inner angle greater than 100 mrad were used for HAADF-STEM observation. An UHV-STEM (TECNAI G^2^) was used to monitor the morphology and particle size of the materials. The samples for UHV-STEM were prepared by dropping a THF suspension of the sample powder onto a commercial TEM grid coated with a collodion film. The sample was thoroughly dried in vacuum prior to observation.

### 3.4. Electrochemical experiment

Electrochemical measurements were performed with a three-electrode system on a HSV-100 electrochemical apparatus. Ag/AgCl (4 M) and a Pt wire were used as the reference and counter electrodes, respectively. A glassy carbon (GC) electrode (PINE, 5 mm diameter) was polished with Gamma Micropolish Alumina (Baikalox, Type 0.05 µm CR) and thoroughly cleaned before use. 4 mg of the catalysts were dispersed in ultrapure water+isopropanol+5% Nafion (v/v/v = 4/1/0.04) with sonication. 45 µl of the suspension was then drop-cast on the cleaned GC electrode and dried at 60°C for 20 min. Prior to the electrochemical measurements, the electrolytes (0.5 M KOH/0.5 M H_2_SO_4_, Fluka) were degassed by bubbling Ar gas for 30 min. Cyclic voltammetry (CV) measurements were performed at a sweep rate of 20 mVs^−1^, with 1 M methanol, ethanol and formic acid present in the electrolyte. Commercial Pt/C NPs (20 wt%, Fuel Store) and Pt_3_Sn/C (Premetek Co.) were used as the control.

Electrochemical active surface area (ECSA) was obtained from the CV of each of the catalysts in 0.5 M KOH/H_2_SO_4_ by measuring the coulombic charge obtained from the area under the Pd-O reduction (Q_o_) curve, assuming that the charge required for the reduction of Pd-O is 0.405 mC.cm^−2^, using the following equation [36–38].

$$ECSA=Q_{o}/0.405mC\;cm^{-2}.$$

For Pt/C and Pt_3_Sn/C, ECSA was calculated using the following equation [39]

$$ECSA=Q_{o}/0.420mC\;cm^{-2}$$

## 4. Results and discussion

**Figure 1.**
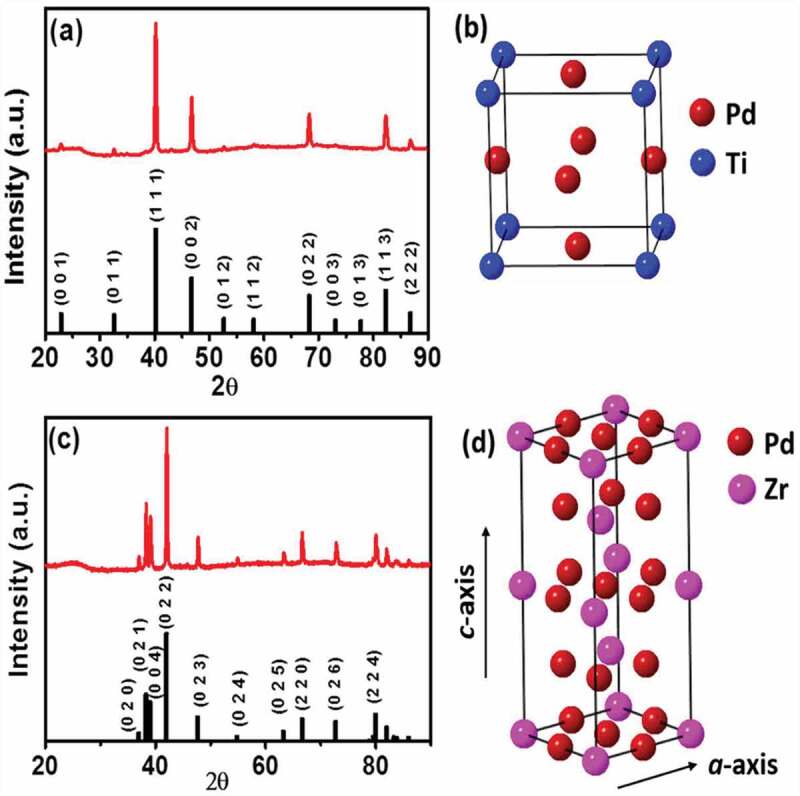
(a) *p*XRD profile of intermetallic Pd_3_Ti/C. (b) Structural model showing the atomic arrangement in Pd_3_Ti. (c,d)*p*XRD profile of Pd_3_Zr/C and structural model of intermetallic Pd_3_Zr. Simulated *p*XRD profiles of intermetallic Pd_3_Ti and Pd_3_Zr are also shown as references.

Figure 1a presents the powder X-ray diffraction (*p*XRD) profile of Pd_3_Ti/C. The *pXRD* profile of Pd_3_Ti shows intense reflection peaks at 40.2°, 46.7°, 68.3° and 82.3°, corresponding to the 111, 002, 022, and 113 reflections of the face-centered cubic (fcc) structure of Pd. However, these peaks are shifted to higher diffraction angles compared to the reflections of pure Pd, which suggests that the smaller atoms have been incorporated into the fcc crystal lattice of Pd. More importantly, the appearance of the less intense peaks at 22.9°, 32.6°, 52.3° and 58.1° clearly indicates the formation of the intermetallic phase. None of these reflections originates from the fcc-type structure instead from an atomically ordered, intermetallic Pd_3_Ti phase. Indeed, the *pXRD* profiles of Pd_3_Ti/C are in good agreement with the simulated *pXRD* pattern of Pd_3_Ti (Cu_3_Au structure, *Pm*$\overset{ˉ}{3}$*m, a* = 3.888 Å) [40]. The atomic arrangements of Pd and Ti in the crystal are shown in Figure 1b. Figure 1c shows the *pXRD* pattern of Pd_3_Zr/C. The *pXRD* profile of Pd_3_Zr/C indicates that the crystal structure of Pd_3_Zr is different from the cubic structure of Pd. The peaks at 37.0°, 38.3°, 39.1°, 54.9°, and 63.2° are assigned to the 020, 021, 004, 024, and 025 reflections, respectively, of an atomically ordered intermetallic Pd_3_Zr. The experimentally observed *pXRD* pattern is fully consistent with intermetallic Pd_3_Zr with a hexagonal crystal structure (space group *P*6_3_/*mmc, a* = 5.6119 Å, *c* = 9.2316 Å, Figure 1d) [41].

**Figure 2.**
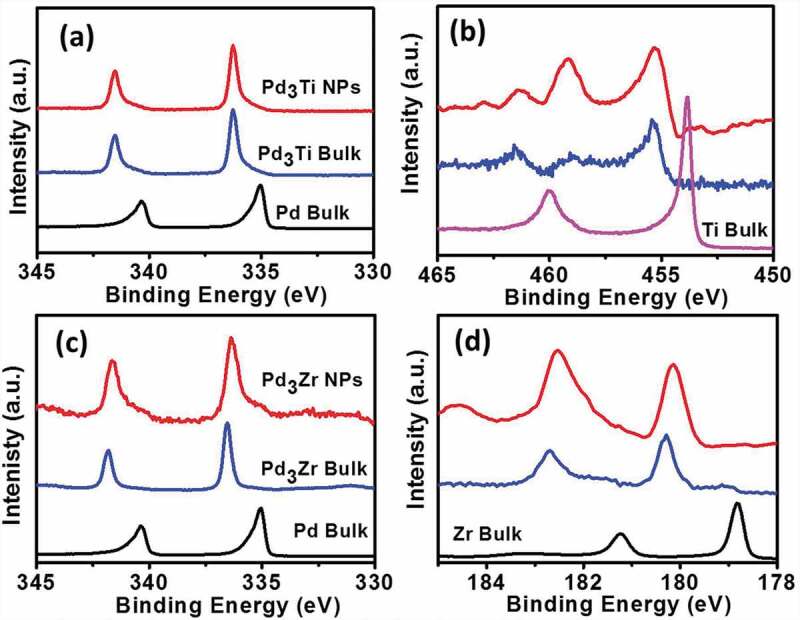
HAXPES spectra of the Pd 3d- (a) and Ti 2p- (b) regions of the Pd_3_Ti NPs and those of the Pd 3d- (c) and Zr 3d- (d) regions of the Pd_3_Zr NPs. HAXPES spectra of the bulk samples of Pd, Ti, Zr, Pd_3_Ti and Pd_3_Zr are shown as references.

HAXPES measurements were used to probe the elemental states of Pd, Ti and Zr in Pd_3_Ti/C and Pd_3_Zr/C. Figure 2a and b show HAXPES spectra of the core levels of Pd and Ti, respectively, in Pd_3_Ti NPs. The binding energy (BE) corresponding to the 3d core level of Pd is shifted to a higher energy, indicating the formation of Pd-Ti bonds in Pd_3_Ti NPs. This is further confirmed by the shift in the BE of the Ti core level to higher energies (Figure 2b). HAXPES analysis also suggests that a finite amount of oxides of Ti was also present in the Pd_3_Ti NPs (Figure S1). A similar trend is observed in the case of Pd_3_Zr. The emissions from the core levels, corresponding to the metallic state of Pd and Zr, are shifted to higher energies, indicative of Pd-Zr bonds in the intermetallic compounds (Figures 2c,d, and S1). The composition of the Pd_3_Zr NPs was calculated from the intensities of the Pd 3*d*_5/2_ and Zr 3*d*_5/2_ emissions as Pd:Zr = 3.0 ± 0.1: 1.2 ± 0.3 [42–45]. The shift of BE to a higher energy arises as a result of the diminished screening of the nuclear charge. The shifts in the BEs of the core levels clearly demonstrate the modification of the electronic structure of Pd, Ti and Zr. The BEs of the core levels of Pd, Ti, and Zr in their respective nanoparticles/bulk samples are tabulated in Tables S1 and S2.

**Figure 3.**
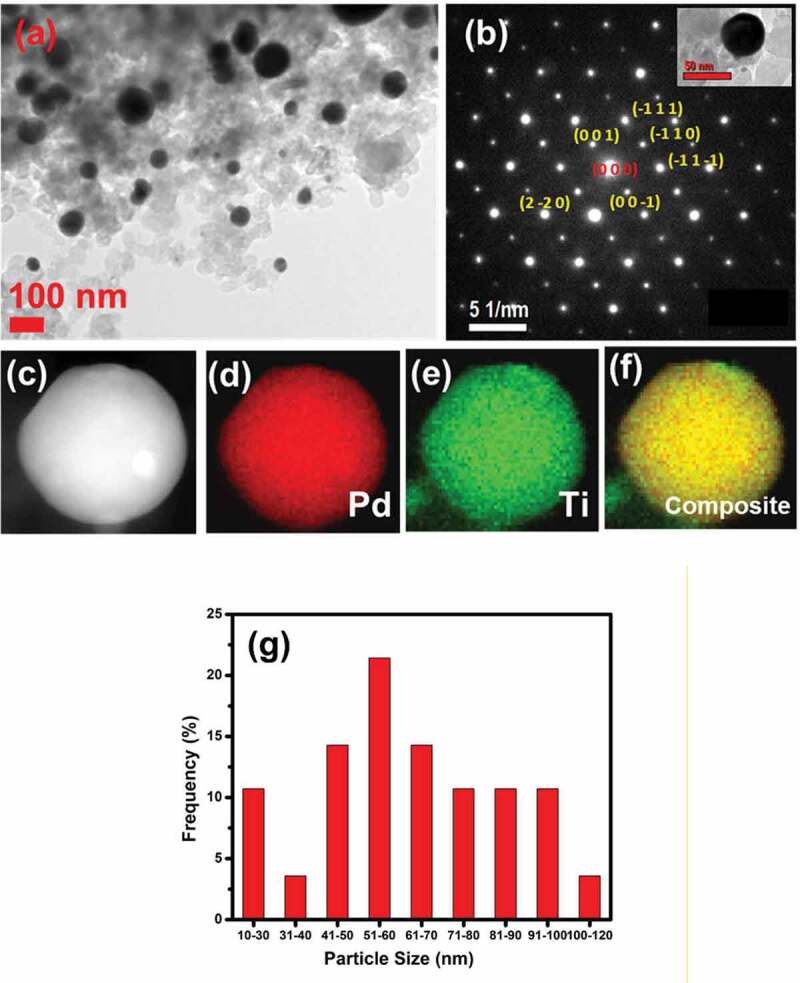
(a)TEM image and (b) selected area diffraction pattern of Pd_3_Ti/C, showing the zone axis of [1 1 0]. (c) STEM image of Pd_3_Ti/C. EDS mapping of (d) Pd, (e) Ti and the composite image for the Pd_3_Ti/C (f). (g) Size distribution of Pd_3_Ti NPs.

**Figure 4.**
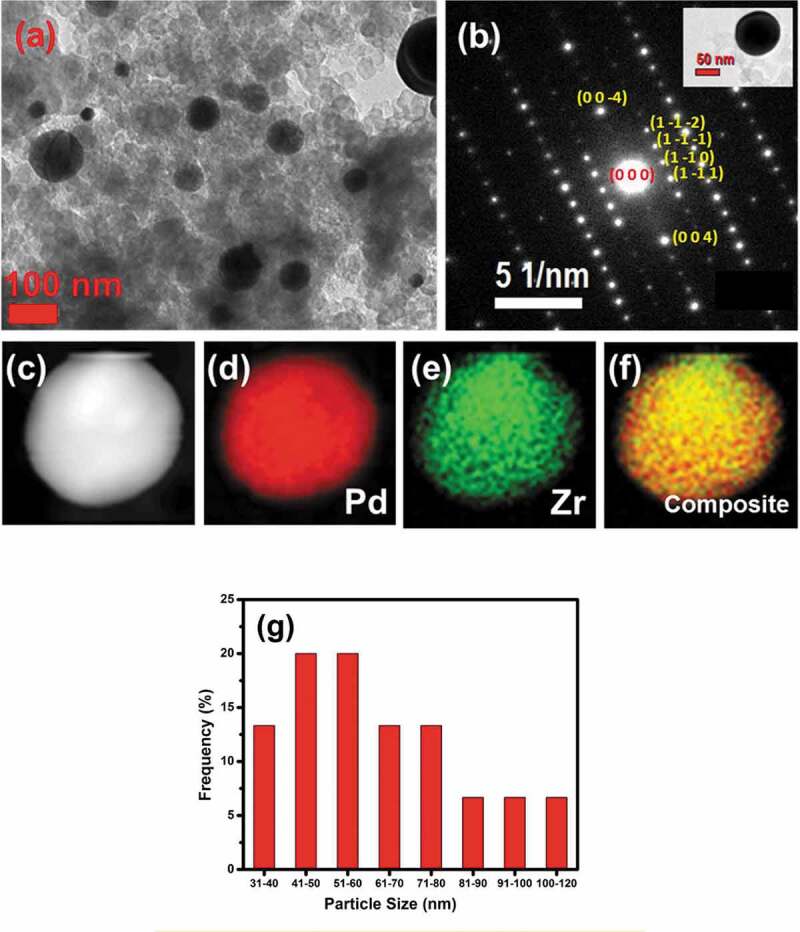
(a)TEM image and (b) selected area diffraction pattern of Pd_3_Zr/C, showing the [1 1 0] zone axis. (c) STEM image of Pd_3_Zr/C. EDS mapping of (d) Pd, (e) Zr and the composite image for the Pd_3_Zr/C (f) are also shown. (g) The statistical distribution of Pd_3_Zr NPs.

Figure 3a shows a TEM image of Pd_3_Ti/C, which consists of spherical particles with a size of 100 nm dispersed on Vulcan carbon. The bigger particles are formed as a result of thermal annealing at high temperature. Selected area electron diffraction (SAED) recorded on an individual nanoparticle is shown in Figure 3b (inset: Pd_3_Ti/C nanoparticle). The obtained pattern fits well with the [1 1 0] zone axis, which further supports the *p*XRD analysis and suggests the formation of Pd_3_Ti intermetallic nanoparticles. Energy-dispersive spectroscopy (EDS) combined with TEM was used to determine the distribution of elements as well as the composition of the nanoparticles. As evident from Figure 3c-f, the elements Pd and Ti are distributed uniformly over the nanoparticles. However, trace amounts of Ti oxides are also observed. Figure 4a shows TEM images of Pd_3_Zr/C nanoparticles. The average size of the particles is estimated to be 100 nm. The Pd_3_Zr nanoparticles are uniformly distributed over the carbon matrix. SAED recorded on the single particles matches the [11 0] zone axis (Figure 3b, inset Pd_3_Zr nanoparticle). The EDS analysis suggests that the distribution of Pd and Zr is uniform within the nanoparticles, with a small amount of oxides on the surface (Figure 4b-e). Compositional analysis with EDS shows that the Pd to Ti/Zr atomic ratio is 3:1 for Pd_3_Ti/C or Pd_3_Zr/C (Figures S2, S3).

**Figure 5.**
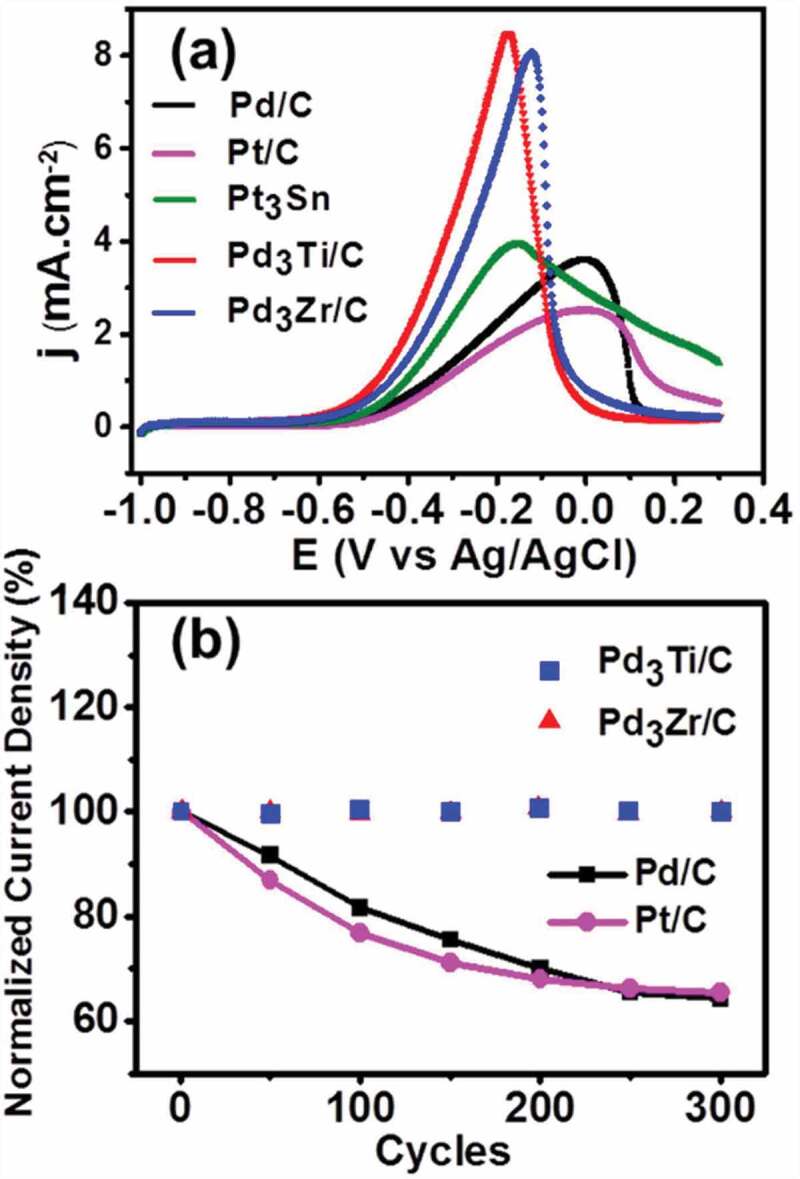
(a) CV curves of methanol oxidation on different catalysts in 1 M methanol + 0.5 M KOH at a scan rate of 20 mVs^−1^. (b) Variation of normalized current density at the peak maximum of different potential cycles from the first to the 300^th^ cycle of methanol oxidation.

The electrocatalytic activity of Pd_3_Ti/C and Pd_3_Zr/C towards methanol and ethanol oxidation reactions (MOR and EOR, respectively) in alkaline media is tested by CV, and compared with that of commercially available catalysts Pd/C (10 wt %), Pt/C (20 wt %), and Pt_3_Sn/C (20 wt %). Figure 5a shows the CV curves obtained for the MOR during the forward scan between a potential range from −1.0 V to 0.4 V.

As is evident from Figure 5a, the specific current density of Pd_3_Ti/C and Pd_3_Zr/C is 3 and 2.5 times higher, respectively, than that of Pd/C. The current density is obtained by normalizing the current with the electrochemical surface area. It is interesting to note that both Pd_3_Ti/C and Pd_3_Zr/C behave similarly, with a slightly higher current density for Pd_3_Ti/C. The peak maximum for the MOR is shifted to a lower potential compared to that of Pd/C, which suggests that the incorporation of Ti or Zr into Pd facilitates the oxidation of methanol. In other words, the oxidation kinetics of methanol on Pd_3_Ti/C and Pd_3_Zr/C are different from those on Pd/C. More importantly, Pd_3_Ti/C and Pd_3_Zr/C exhibit a higher specific activity (1.8 times for Pd_3_Ti/C and 1.6 times for Pd_3_Zr/C) in comparison with that of Pt/C. Compared with Pd/C and Pt/C, the lower oxidation peak potential and higher oxidation current density indicate that Pd_3_Ti/C and Pd_3_Zr/C can be considered as promising candidates for the MOR. The long-term stability and activity of Pd_3_Ti/C and Pd_3_Zr/C for the MOR was tested by chronoamperometry at −0.2 V. The intermetallic compounds show a higher current density compared to that of Pd/C for 3600 s (Figure S4). The reproducible operation of the catalysts was further verified by accelerated durability tests (ADT) at a scan rate of 90 mVs^−1^. The durability was tested by comparing the peak current density for different potential cycles (Figures 5b, S5). As is evident from Figure 5b, the current density remains virtually the same even after 300 potential cycles for Pd_3_Ti/C and Pd_3_Zr/C. However, Pd/C and Pt/C lose ~40% to 50% of the initial current density after 300 cycles (Figure S5).

**Figure 6.**
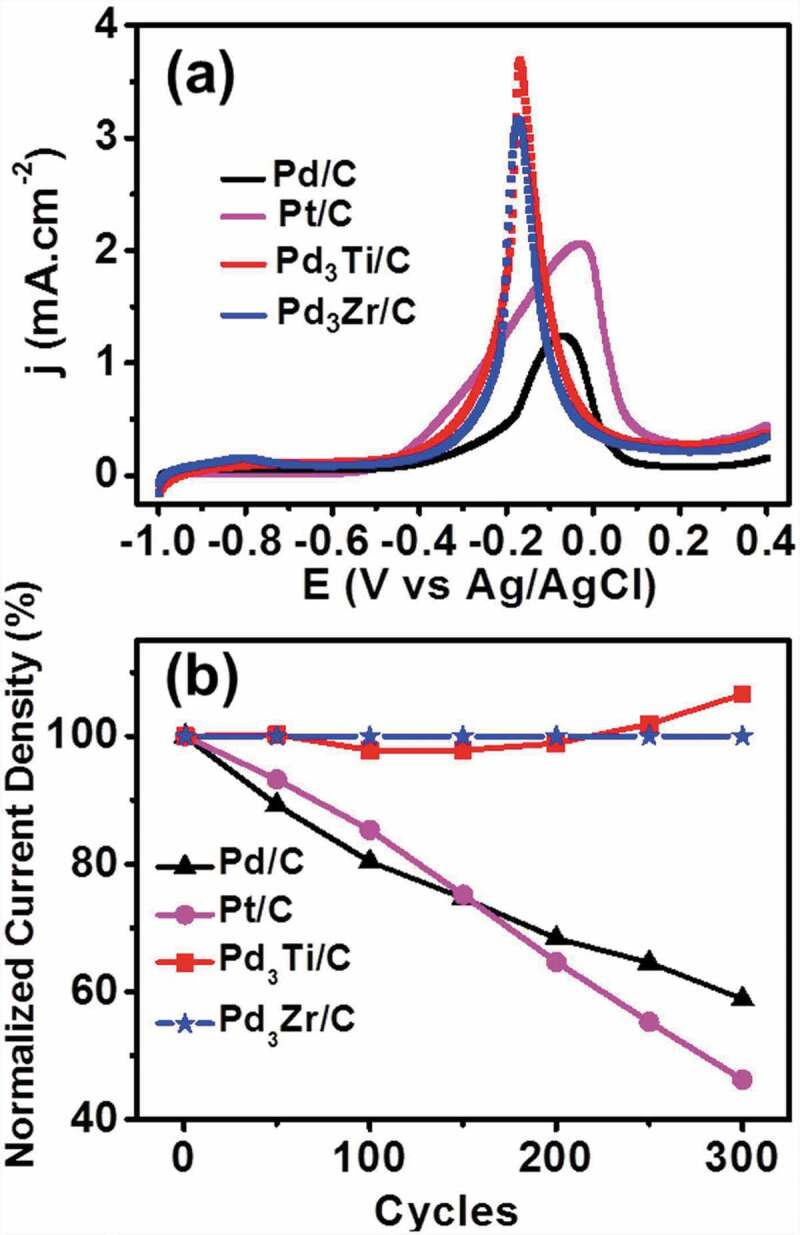
(a) CV curves of ethanol oxidation on different catalysts in 1 M ethanol + 0.5 M KOH at a scan rate of 20 mVs^−1^. (b) Variation of normalized current density at the peak maximum of different potential cycles from the first to the 300^th^ cycle of ethanol oxidation.

Figure 6a shows the CV curves of the EOR on different catalysts. The specific current density for Pd_3_Ti/C and Pd_3_Zr/C is 2.35 and 2.25 times higher, respectively, than that of Pd/C. In addition, the onset potential for Pd_3_Ti/C (−0. 574 V) and Pd_3_Zr/C (−0. 521 V) is shifted to a significantly lower potential in comparison with that of Pd/C (−0. 480 V). Importantly, the oxidation peak potential of the EOR shifts by −0.175 V for Pd_3_Ti/C and −0.125 V for Pd_3_Zr/C, in comparison with that of Pd/C (0 V). It should be noted that the activity of Pd_3_Ti/C and Pd_3_Zr/C towards the EOR is remarkably higher than that of Pt/C and Pt_3_Sn/C. Considering the onset potential, current density, and oxidation peak potential, Pd_3_Ti/C is found to be the best catalyst for the EOR. Note that the oxidation peaks for the MOR and EOR over the Pd_3_Ti- and Pd_3_Zr catalysts were narrow and sharp compared to those for the control materials, Pd/C, Pt/C or Pt_3_Sn/C, reflecting that the surface Pd atoms are surrounded by the Ti- and Zr-atoms to act as an isolated adsorption centre for the reaction intermediate, CO.

Chronoamperometry measurements at −0.2 V show that Pd_3_Ti/C and Pd_3_Zr/C are superior to Pd/C, exhibiting a higher current density for 600 s (Figure S6), indicating a higher tolerance of Pd_3_Ti/C and Pd_3_Zr/C to the carbonaceous species generated during ethanol oxidation. The durability of the catalysts in the EOR was further tested with ADT at a scan rate of 90 mVs^−1^ (Figure 6b). The current density of Pd_3_Ti/C and Pd_3_Zr/C increases with an increasing number of potential cycles and then remains constant, even after 300 ADT cycles. However, the activity of Pd/C and Pt/C is decreased by 40% after 300 cycles (Figure S7). Therefore, the above results demonstrate that Pd_3_Ti/C and Pd_3_Zr/C show improved catalytic activity and stability in reproducible operation for the EOR.

**Figure 7.**
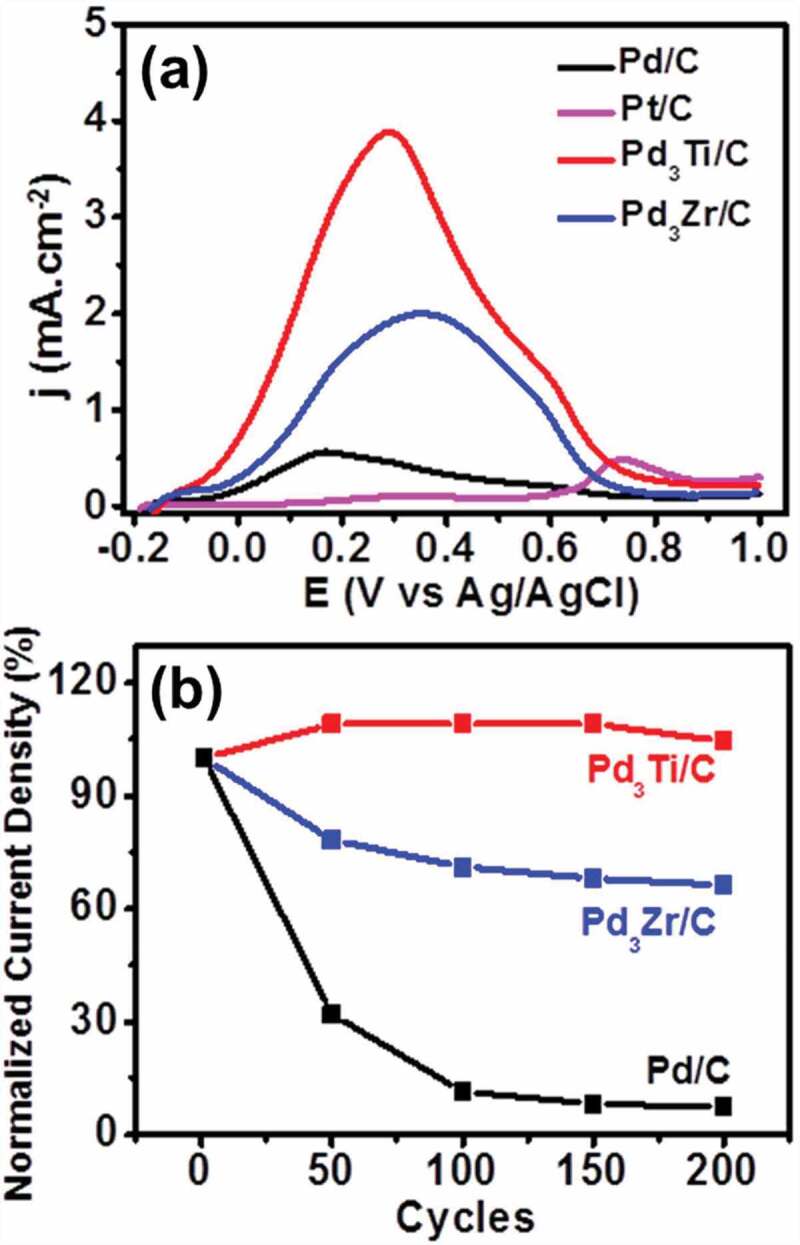
(a) CV curves of formic acid oxidation on different catalysts in 1 M formic acid + 0.5 M H_2_SO_4_ at a scan rate of 20 mVs^−1^. (b) Variation of normalized current density at the peak maxima of different potential cycles from the first to the 200^th^ cycle of formic acid oxidation.

We further tested the activities of the catalysts towards formic acid electro-oxidation (FAEO) in an acidic medium (Figure 7a). Pd-based catalysts are known to facilitate the oxidation of formic acid through the dehydrogenation pathway. The peak current intensity for Pd_3_Ti/C and Pd_3_Zr/C is 6.8 and 3.5 times higher, respectively, than that of Pd/C. The onset potential of Pd_3_Ti/C (−0.118 V) is much more negative than that of Pd_3_Zr/C (−0.04 V) and Pd/C (−0.07 V).

The ADT experiments finally suggest that Pd_3_Ti/C exhibits very good tolerance to repeated operation towards formic acid oxidation. For Pd_3_Ti/C, the current density initially increases with the number of cycles and remains constant even after 200 cycles (104%). However, the activity of Pd_3_Zr/C is decreased with an increasing number of cycles (35% loss) after 200 cycles. On the other hand, Pd/C loses almost 90% of its initial activity within even 100 potential cycles, which suggests that alloying of Pd with Ti/Zr improves the stability of the catalysts (Figure 7b). The superior stability of Pd_3_Ti/C to Pd_3_Zr/C may be attributed to the chemical stability of titanium oxides (TiO*_x_*) layers on the catalyst surface (Figure S1), which can inhibit surface segregation or dealloying in acidic electrolytes.

The *d*-band centers of Pd_3_Ti- and Pd_3_Zr NPs were calculated as −3.47 eV and −3.71 eV, respectively. These values are lowered significantly compared to that of pure Pd, which is close to −3.10. More importantly, the *d*-band centers of Pd_3_Ti- and Pd_3_Zr NPs are even lower than that of Pt, −3.31 eV (Figure S8). The lowered *d*-band center of Pd_3_*X* can alter hybridization strength of one of the major reaction intermediates of FAEO, MOR and EOR, carbon monoxide that works a catalytic poison, which may result in the improved electrocatalytic performances.

## 5. Conclusion

We have developed two Pd-based inter-metallic nanocrystals with the early d-metals Ti and Zr. The Pd_3_Ti/C and Pd_3_Zr/C catalysts were characterized in detail using *pXRD*, TEM and EDS. These newly developed catalysts exhibit enhanced catalytic activity towards methanol and ethanol electro-oxidation in an alkaline medium and formic acid electro-oxidation in an acidic medium, compared to commercially available catalysts.

## Supplementary Material

Supplemental Material

## Supporting information

Synthesis, characterization (*p*XRD, HAXPES, TEM) and electrochemical data.
