# Supplementary material for: Intermetallic Pd3X (X= Ti and Zr) nanocrystals for electro-oxidation of alcohols and formic acid in alkaline and acidic media
Source: Sci Technol Adv Mater. 2020 Aug 24;21(1):573–83. doi: 10.1080/14686996.2020.1789437 (PMC7476510; doi:10.1080/14686996.2020.1789437)
Supplement: Supplemental Material [file TSTA_A_1789437_SM1324.pdf]

Supporting information for

Intermetallic Pd_3X (X= Ti and Zr) nanocrystals for Electro-oxidation of Alcohols and Formic Acid in Alkaline and Acidic medium

Rajesh Kodiyath,*[,a] Gubbala V. Ramesh,*[,a,b] MaidhilyManikandan,[a] Shigenori Ueda,[c] Takeshi Fujita[d] and Hideki Abe*[,a]

[a] National Institute for Materials Science, 1-1 Namiki, Tsukuba, Ibaraki 305-0044, Japan.

b Department of Chemistry, Chaitanya Bharathi Institute of Technology (A), Gandipet, Hyderabad – 500075, Telangana, India.

[c] Synchrotron X-ray Station at SPring-8, National Institute for Materials Science, 1-1-1 Kouto, Sayo, Hyogo, 679-5148, Japan.

[d] School of Environmental Science and Engineering, Kochi University of Technology, 185 Miyanouchi, Tosayamada, Kami City, Kochi 782-8502, Japan.

Email: rajeshkodiyath@gmail.com, venkataramesh_chm@cbit.ac.in, abe.hideki@nims.go.jp

Information Classification: General

Synthesis:

Reagents used for the synthesis:Anhydrous Palladium (II) acetate, (Aldrich), anhydrous Titanium(IV) chloride tetrahydrofuran complex ($TiCl_4$.2THF,Aldrich,97%), Zirconium (IV) chloride ($ZrCl_4$, Aldrich, 99%), Diglyme (anhydrous, 99.8%, Aldrich),$LiBH(C_2H_5)_3$(super-hydride, 1M in THF, Aldrich), Hexane (anhydrous, 95%, Aldrich) and acetonitrile (99.8%, Aldrich), sodium metal (Aldrich) and naphthaline (Fisher).

Synthesis of Pd_3Ti/CNPs: Intermetallic Pd_3TiNPs were synthesized by co-reduction of metal precursors in diglyme.Palladium (II) acetate (28.86 mg, 0.13 mmol) and $TiCl_4$.2THF (15 mg, 0.035 mmol) were weighed and transferred to a round bottom flask containing strong reducing agent sodium naphthalide.54 mg of Vulcan carbon is added to the reaction mixture.The reaction mixture wasthen transferred to a reaction vessel and heated the reaction mixture at 200 ^{o}Cin an oil bath for 2h under an argon pressure of 0.5 MPa. The product was then transferred to a centrifuge tube under argon atmosphere. The precipitate was separated from diglyme by centrifuging at 6000 rpm for 5 min. The product was washed several times with hexane and acetonitrile to remove the byproducts. The product was driedunder vacuum for 1h. The washing solvents were carefully chosen so that interaction with the oxophilic metal is minimized and hence leaching or dissolution of the metal resulting in non-uniform composition. The as prepared product was annealed at 1000 °C for 15h under vacuum to achiev the desired intermetallic Pd_3Ti phase.

Synthesis of Sodium naphthalide: Sodium naphthalide solution was prepared by dissolving 22.9 mg of metallic sodium and 129.4 mg of naphthalene in dry diglyme. The reaction mixture was stirred for overnight under argon atmosphere.

Synthesis of Pd_3Zr/C NPs: Intermetallic Pd_3Zr NPs were synthesized by co-reduction of metal precursors in diglyme.Palladium (II) acetate (72.9 mg, 0.32 mmol) and $ZrCl_4$ (26.5 mg, 0.113 mmol) were weighed in a custom made vessel. 30 ml of diglyme was then added to the vessel stirred the mixture for 20 min to dissolve the reactants. 1ml of super-hydridewas then added to the reaction mixture and heated the reaction mixture at 200 ^{o}C in an oil bath for 2h under an argon pressure of 0.5 MPa. The product was then transferred to a centrifuge tube under argon atmosphere. The precipitate was separated from diglyme by centrifuging at 6000 rpm for 5 min. The product was washed several times with hexane and acetonitrile to remove the byproducts. The product was dried under vacuum for 1h. The as prepared product was then mixed with Vulcan carbon to form Pd_3Zr/C. The as prepared product Pd_3Zr/C was annealed at 1000 °C for 15h under vacuum to obtain intermetallic Pd_3Zr/C.

Synthesis of bulk Pd_3Ti and Pd_3Zr.

Polycrystalline bulk samples of intermetallic $TaPt_3$ were synthesized with an arc furnace in a pure Ar atmosphere (99.9999 %). Prior to the synthesis, the arc furnace was evacuated to a vacuum level lower than 10 mPa and back-filled with pure Ar. All the starting materials were purchased from Furuya Kinzoku Co. An aliquot of 1 g of Pd powder (99.9 %) was pelletized with a stainless-steel die and melted with the arc furnace into an ingot. Ti/Zr (ingot, 99 %) was used as received. The ingots of Ti/Zr and Pd were weighed such that the molar ratio was

Ti/Zr:Pt = 1:3 and melted together in the arc furnace to obtain the desired intermetallic Pd_3Ti/Pd_3Zr. The final product was finally annealed in vacuum at 1000 °C for 72 h.

Characterization:

Powder X-ray diffractometry (*p*XRD): The *p*XRD measurements were performed using Cu Kα radiation (λ =0.15418 nm) with an increment of 0.02 degrees in a range of diffraction angles from 20 to 100 degrees. An obliquely finished Si crystal (non-reflection Si plate) was used as a sample holder to minimize the background.

Hard X-ray photoemission spectroscopy (HX-PES): HX-PES measurements were performed using X-rays with a photon energy of 5.95 keV, at the undulator beamline BL15XU of SPring-8, Japan. Samples for HX-PES measurements were prepared by mixing the sample solution (in THF) with carbon black (Vulcan XC-72, Cabot Co. Ltd.) to avoid charging effects. 10 µl of the sample was dropped onto carbon substrates (Nilaco Co., Ltd.) and dried under vacuum. The core-level states of the samples were examined at room temperature in UHV using a hemispherical electron energy analyzer (VG SCIENTA R4000). The total energy resolution was set to 220 meV. The binding energy was referenced to the Fermi edge of an Au thin film.

Transmission electron Microscopy: We used a 200 kV transmission electron microscope (TEM and/or STEM, JEM-2100F, JEOL) equipped with two aberration correctors (CEOS GmbH) for the image- and probe-forming lens systems and an X-ray energy-dispersive spectrometer (JED-2300T, JEOL) for compositional analysis. Both the aberration correctors were optimized to realize the point-to-point resolutions of TEM and scanning transmission electron microscopy (STEM) as 1.3 and 1.1 Å, respectively. A probe convergence angle of 29 mrad and a high-angle annular-dark-field (HAADF) detector with an inner angle greater than 100 mrad were used for HAADF-STEM observation. An ultra-high-vacuum STEM (UHV-STEM; TECNAI G^2) was used to perform microscopic observation of the morphology and particle size of the materials. The samples for UHV-STEM were prepared by dropping a THF suspension of the sample powder onto a commercial TEM grid coated with a collodion film. The sample was thoroughly dried in vacuum prior to observation

.

Electrochemical Experiment: Electrochemical measurements were carried out with a three electrode system on HSV-100 electrochemical apparatus. Ag/AgCl (4 M) and a Pt wire were used as the reference and counter electrodes respectively. Glassy carbon (GC) electrode (PINE, 5 mm diameter) was polished with Gamma Micropolish Alumina (Baikalox, Type 0.05 µm CR) and thoroughly cleaned before its use. 4 mg of the catalysts were dispersed in ultrapure water+isopropanol+5% Nafion (v/v/v =4/1/0.04) with sonication. 45 µl of the suspension wasthen drop cast on the cleaned GC electrode, and dried at 60 °C for 20 min. Prior to the electrochemical measurements, the electrolytes (0.5 M KOH/0.5 M H_2SO_4, Fluka) weredegassed by bubbling Ar gas for 30 min. CV measurements were performed at a sweep rate of 20 mVs^{-1} with 1 M methanol, ethanol and formic acid present in the electrolyte.

Information Classification: General

ECSA was obtained from the CV of each of the catalysts in 0.5 M KOH/H_2SO_4 by measuring the columbic charge obtained from the area under the Pd-O reduction (Qo) curve assuming that the charge required for reduction of Pd-O is 405 µC cm^{-2} using the following equation.[1]

ECSA= Q_o/0.405 mC cm^{-2}.

For Pt/C and Pt_3Sn/C, ECSA was calculated using the following equation

ECSA= Q_o/0.420 mC cm^{-2}

Information Classification: General

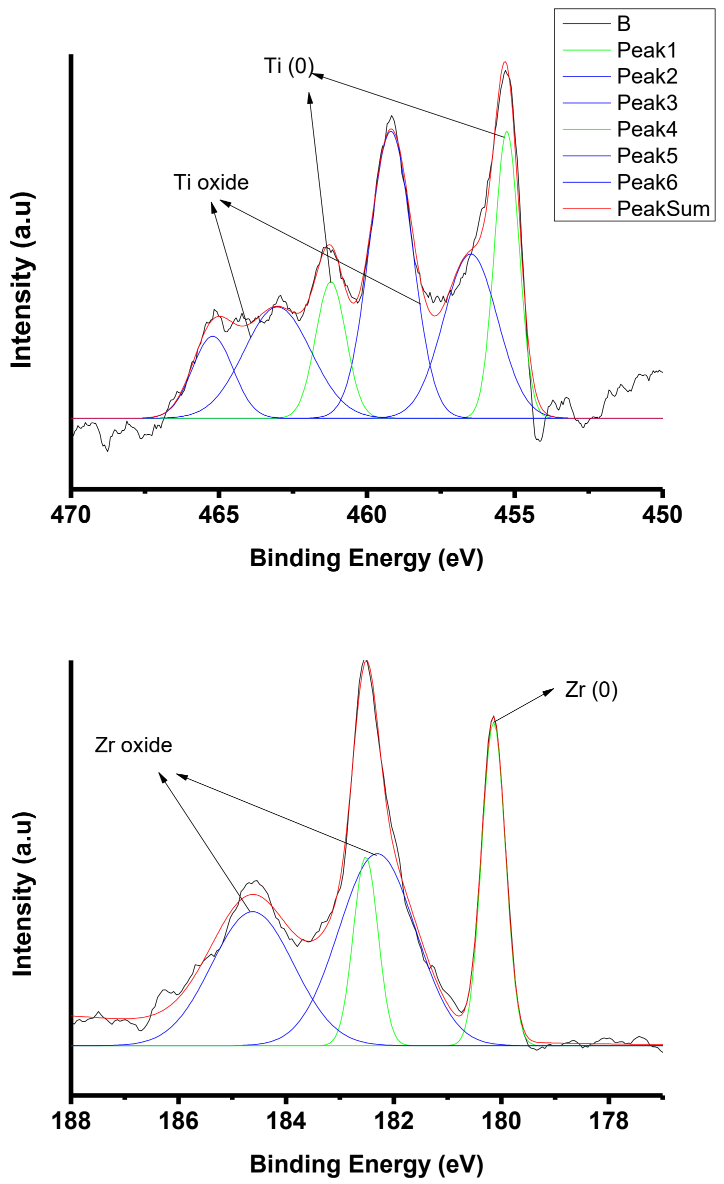

Figure S1. (a) Deconvoluted HX-PES spectra of Ti 2p collected on Pd_3Ti/C. (b) Deconvoluted HX-PES spectra of Zr 3d collected on Pd_3Zr/C

Information Classification: General

Sample	Ti (0) Core level (eV)		TiOx Core level (eV)	
	Ti 2p3/2	Ti 2p1/2	Ti 2p3/2	Ti 2p1/2
Pd_3Ti NPs	455.3	461.2	459.2	465.2
	Zr (0) Core level (eV)		ZrOx Core level (eV)	
	Zr 3d5/2	Zr 3d3/2	Zr 3d5/2	Zr 3d3/2
Pd_3Zr NPs	180.14	182.5	182.3	184.6

Table S1: Binding energies of Ti, Zr and Pd core levels of Pd_3Ti and Pd3Zr NPs.

Information Classification: General

**Sample**	**Pd Core level (eV)**	
	**Pd $3d_{5/2}$**	**Pd $3d_{3/2}$**
Pd Bulk	335.01	340.37
Pd_3Ti/C	336.25	341.53
Pd_3Zr/C	336.40	341.64

Table S2: Binding energies of Pd core levels of Pd bulk, Pd_3Ti/C and Pd3Zr/C.

Information Classification: General

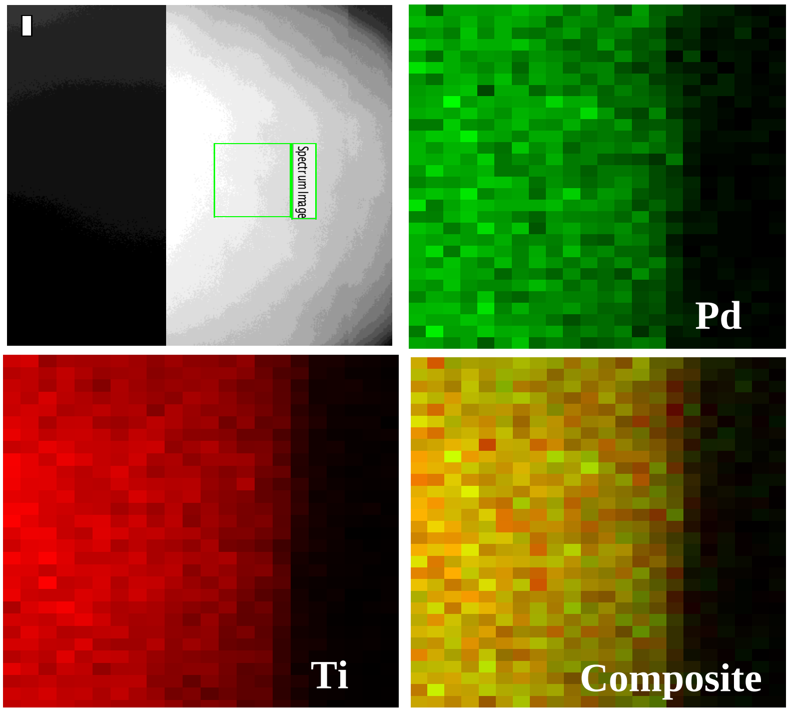

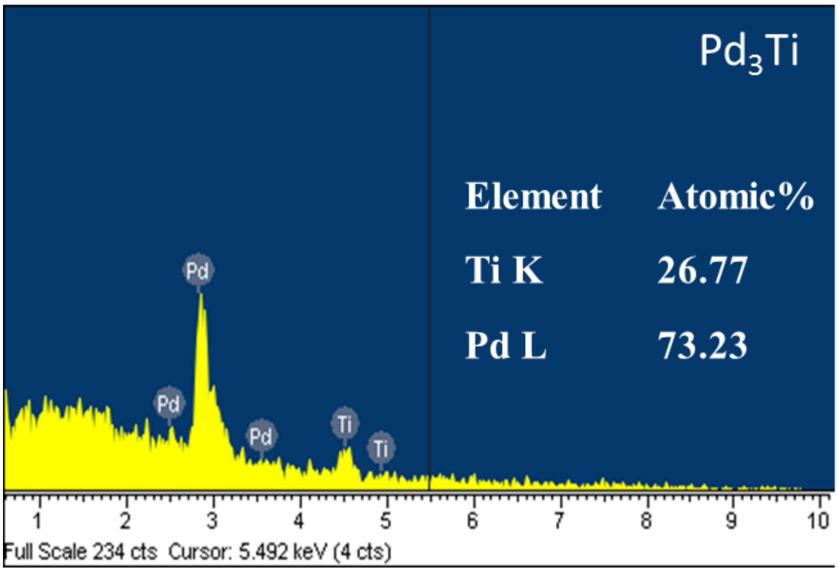

Figure S2.STEM image and the corresponding elemental mapping images of Pd_3Ti/C. EDS spectrashowing Pdto Tiatomic ratio.

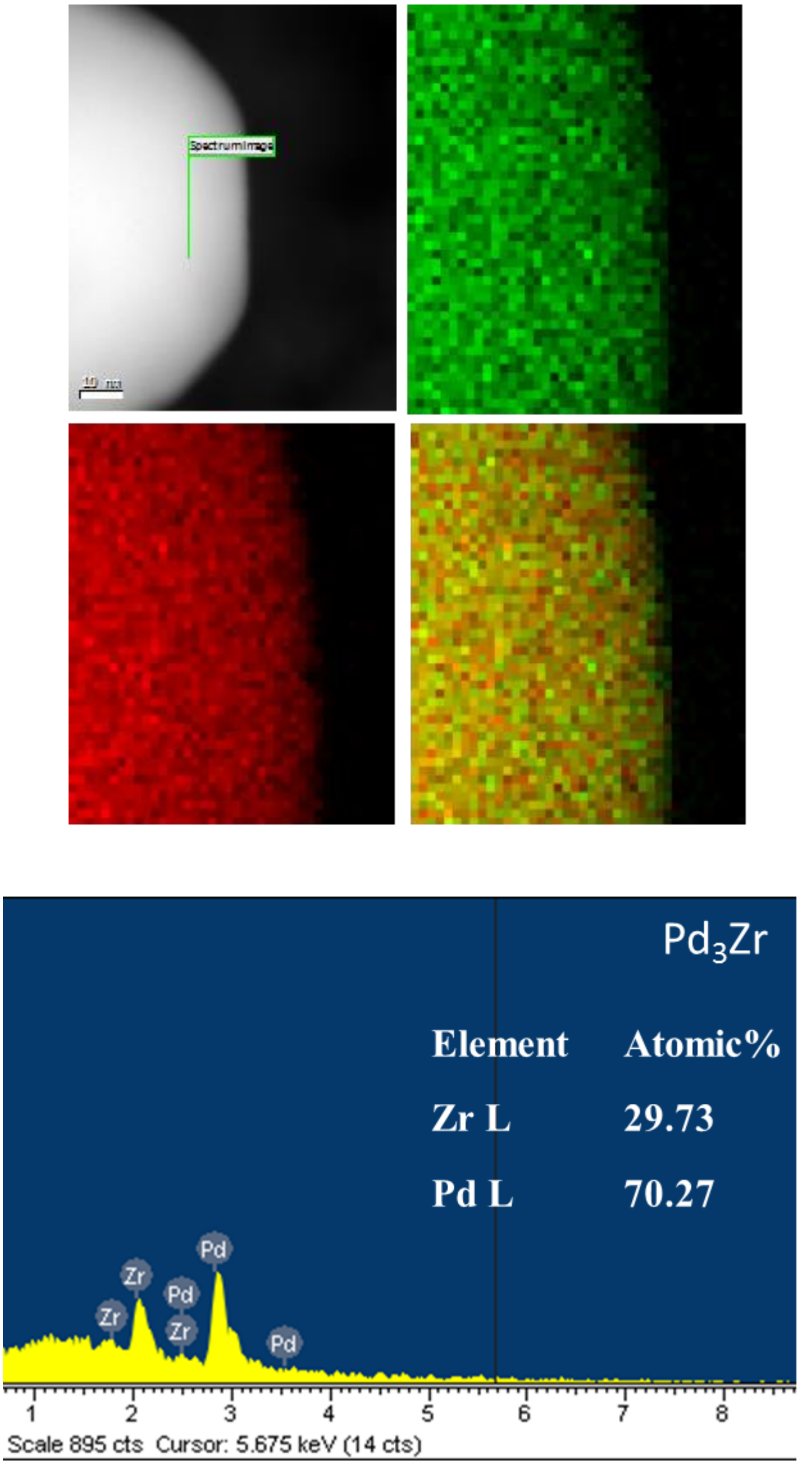

Figure S3. STEM image and the corresponding elemental mapping images of Pd_3Zr/C. EDS spectra showing Pd to Zr atomic ratio.

Information Classification: General

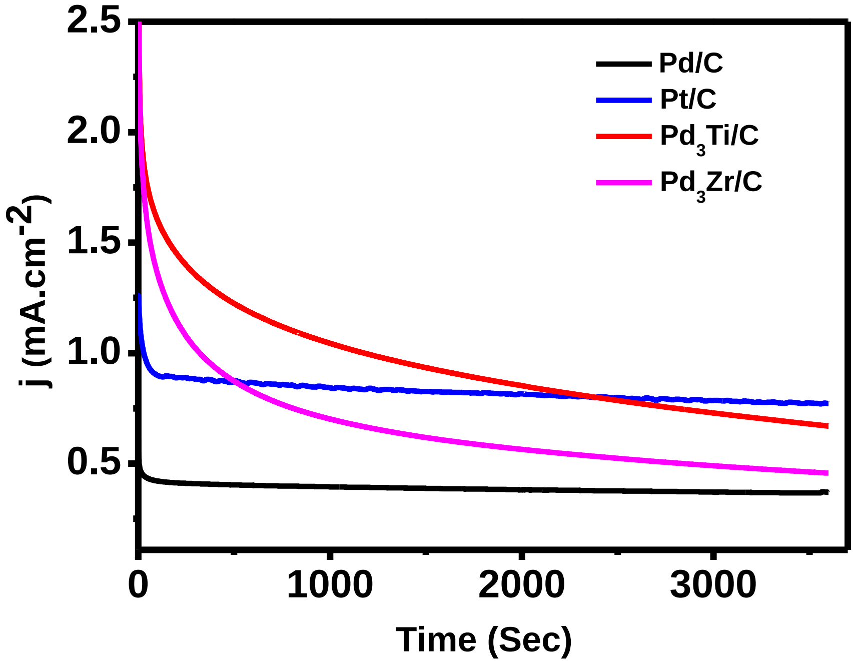

Figure S4. Chronoamperometry recorded for the MOR on various catalysts at -0.2 V Vs Ag/AgCl in 1 M Methanol + 0.5 M KOH

Information Classification: General

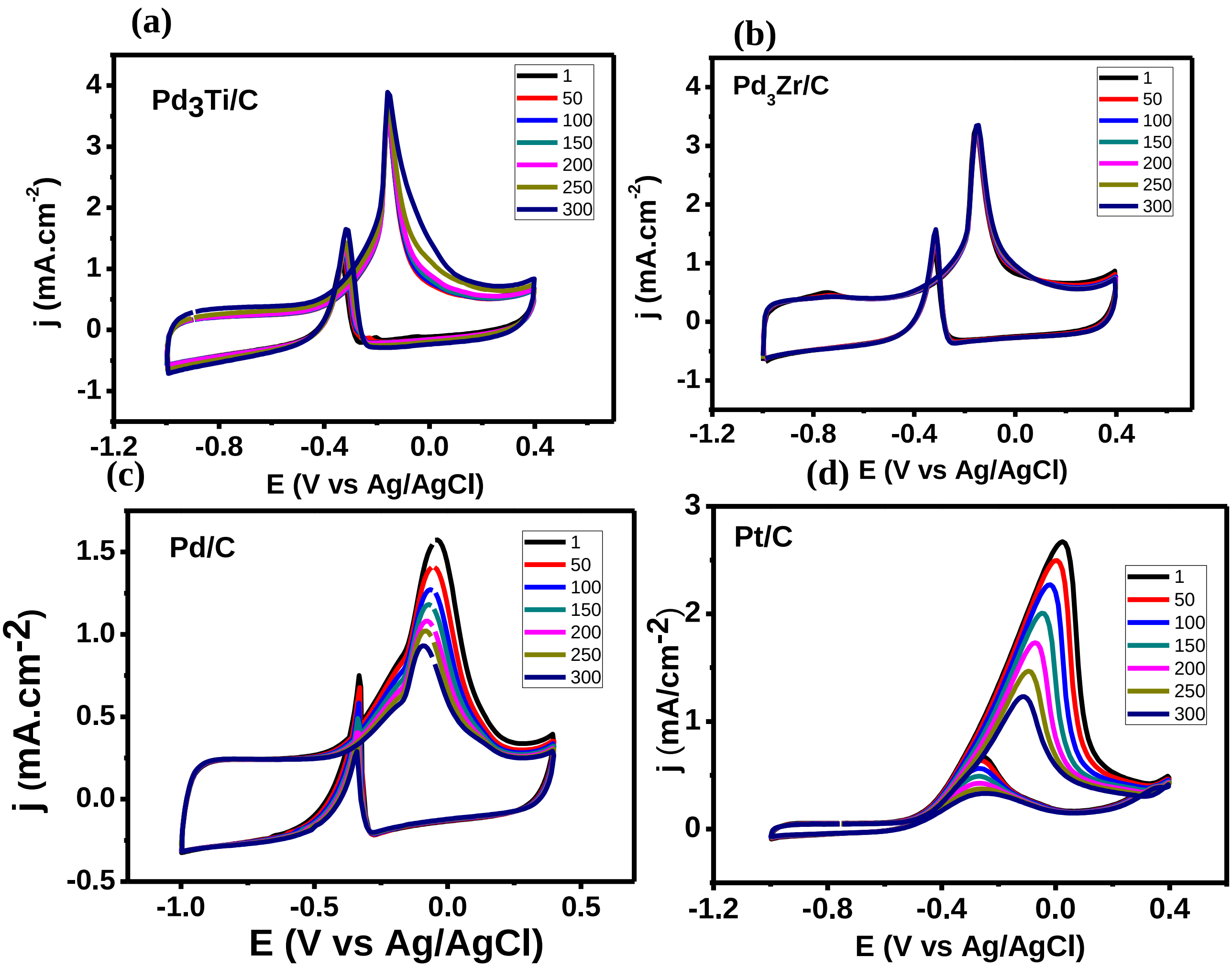

Figure S5. CVs from first to 300th cycles for (a) Pd_3Ti/C (b) Pd_3Zr/C (c) Pd/C and (d) Pt/C in 1.0 M Methanol + 0.5 M KOH at 90 mVs^{-1}.

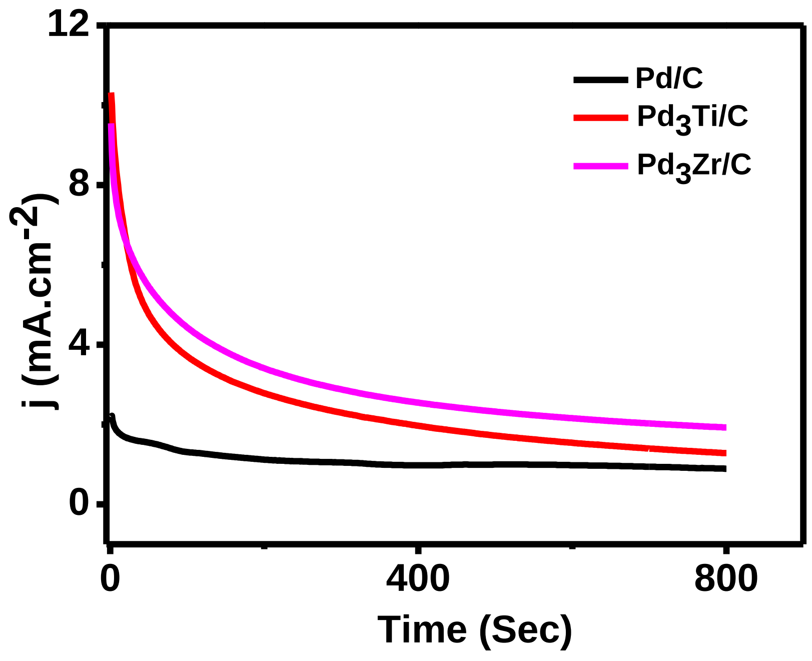

Figure S6. Chronoamperometry recorded for the EOR on various catalysts at -0.2 V Vs Ag/AgCl in 1 M Ethanol + 0.5 M KOH

Information Classification: General

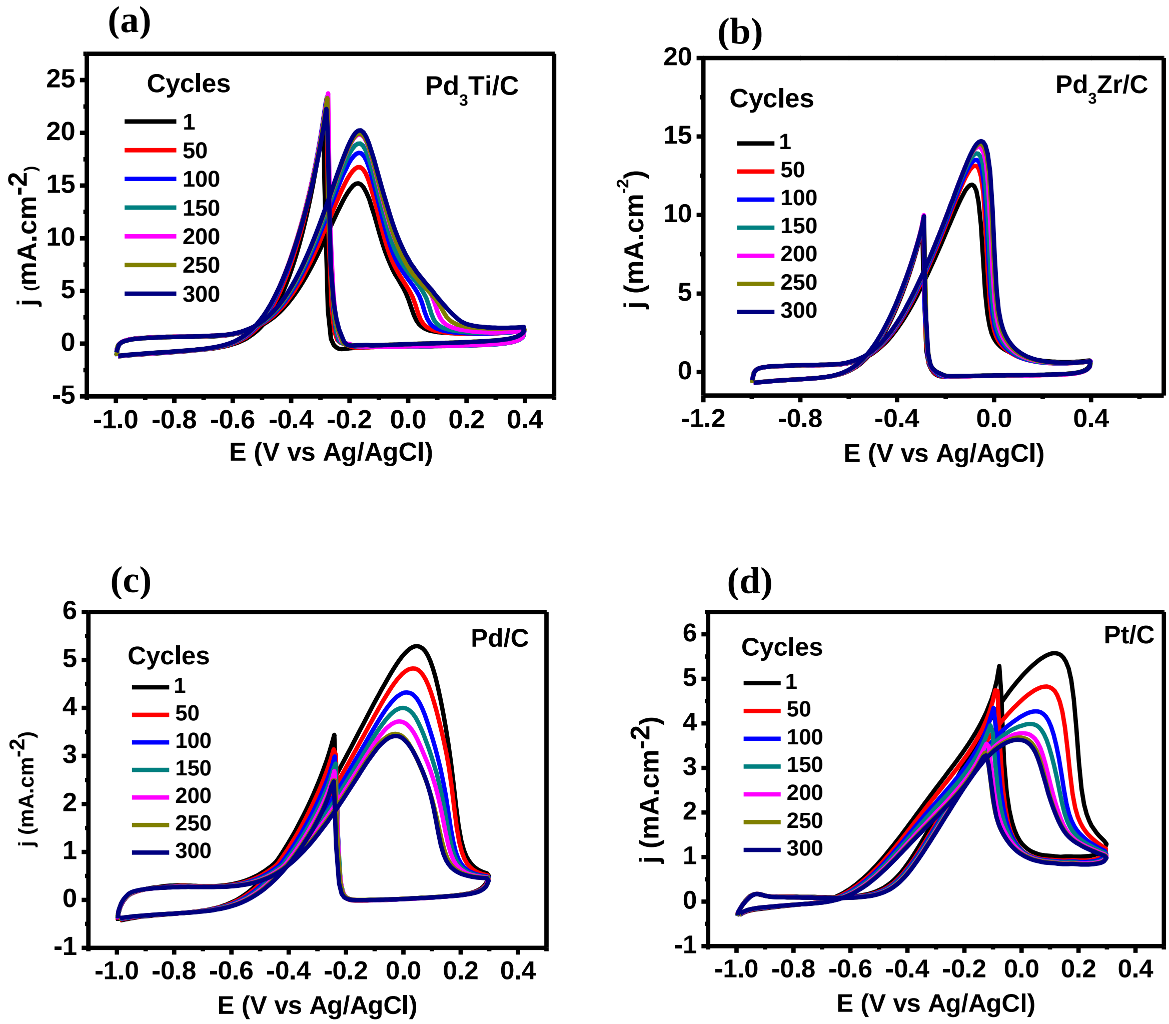

Figure S7. CVs from first to 300th cycles for (a) Pd_3Ti/C (b) Pd_3Zr/C (c) Pd/C and (d) Pt/C in 1.0 M Ethanol + 0.5 M KOH at 90 mVs^{-1}.

Information Classification: General

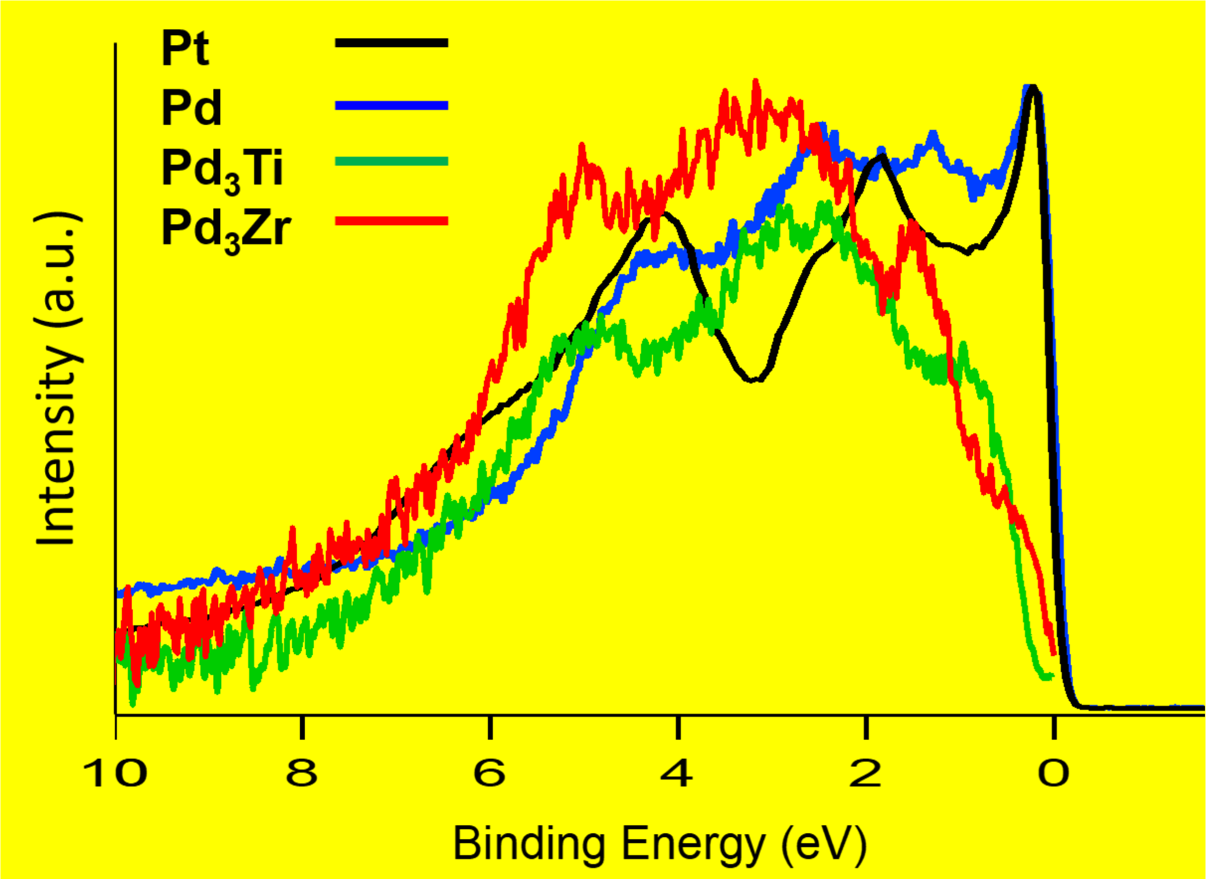

Figure S8. HX-PES spectra in the valence region of Pd_3Ti/C and Pd_3Zr/C. The HX-PES spectra for bulk Pt is shown as the reference.

The center-of-gravity of the valence *d*-band (*d*-band center) for the materials was calculated from these HX-PES spectra using the following formula:

$$d - bandcenter = \int_{-8\,eV}^{0\,eV} (BindingEnergy(E) \times Intensity(E))\, dE \Big/ \int_{-8\,eV}^{0\,eV\,(Ef)} Intensity(E)\, dE$$

1. (a) Zhang, Z.; More, K. L.; Sun, K.; Wu, Z.; Li, W. *Chem. Mater*. **2011**, *23*, 1570-1577.(b) Jiang, L.; Hsu, A.; Chu, D.; Chen, R. *J. Electrochem.Soc*. **2009**,*156(3),* B370-B 376. (c) Singh, R. N.; Singh, A.; Anindita, Int. *J. Hydrog. Enegy*, **2009**, *34*, 2052.

Information Classification: General
